# The Physiology of the Bile and Gall-Bladder, and the Etiology of Cholelithiasis*Abstract of paper read at meeting of Richmond Academy of Medicine and Surgery.

**Published:** 1895-09

**Authors:** H. H. Levy

**Affiliations:** Richmond, Va.


### THE PHYSIOLOGY OF THE BILE AND GALL-BLADDER, AND THE ETIOLOGY OF CHOLELITHIASIS.*

By H. H. LEVY, M.D.,
RICHMOND, VA.

The color of the bile varies, as does its consistency. When fresh it is slightly viscid, due to the mucus of the bladder and ducts. Normally, it is of a neutral reaction, but may be alkaline, or even acid. The important constituents are: 1. Mucus, prone to decompose and cause alterations in the chemical construction of the bile. 2. Bile salts, taurocholate and glycocholate of sodium. 3. Pigments, the chief being bilirubin and biliverdin, the former often occurring in combination with alkalies. It is similar to hematoidin. 4. Choles-

---

*Abstract of paper read at meeting of Richmond Academy of Medicine and Surgery.

terin in small amounts, characterized by being levorotatory and occurring in rhombic plates that seem to have one corner broken off. 5. Diastasic ferment. 6. Traces of urea. 7. Inorganic constituents—salts as found in most other secretions, and a considerable amount of $CO_2$ in fresh bile, either free or in combination.

The small amount of cholesterin is noteworthy. The secretion is not a mere filtration, but a product of the liver cells, always going on, greater at times than at others. It does not pass immediately into the intestine when digestion is not taking place, but regurgitates to the gall-bladder, where it is kept until needed. The quantity secreted in twenty-four hours is about 1–14000 of the body weight, and the period of greatest flow into the intestine is about three or four hours after the ingestion of food. Circumstances influencing secretion are: 1. Food. Nitrogenous increases it more than vegetable, while fatty foods have no effect. 2. Water in large amounts increases the quantity but lowers the specific gravity. 3. Other things equal, an increased blood supply increases the quantity, and *vice versa.* 4. Any condition increasing disintegration of the red corpuscles increases the quantity of bile.

The flow is influenced by: 1. The *vis a tergo.* 2. Descent of the diaphragm pressing on the liver in inspiration. Negative pressure produced by inspiration also aids it. 3. Contraction of the muscular fibers of the bladder and ducts. 4. Stimulation of the cord, as by passage of food into the stomach and duodenum. In connection, a practical point to note is, a small amount of resistance to outflow is sufficient to cause stagnation of the bile.

*Disposal of the Bile.*—The water aids the maintenance of the softness of the feces. Mucus passes out unchanged. The pigments do not appear in their own forms in the excretions, but as hydrobilirubin, urobilin, and stercobilin. The meconium of the fetus contains the pigments unchanged. The bile salts are mainly reabsorbed. Cholesterin and lecithin are found in the feces.

*Composition of Gall-stones.*—Cholesterin constitutes by far the greatest number, but all do not contain it, and often it is mixed with fatty or saponaceous matters or pigments. Some are composed of bilirubin (mostly in combination with calcium), in strata or masses of cholesterin. Hydrobilirubin may alone form them, as also may glycocholate and taurocholate of calcium. Many are made up of fatty acids and soaps. Mucus and epithelium occasionally constitute small stones, or the nuclei of larger ones. Sometimes they are formed of the oxides of the heavy metals, with, occasionally, nuclei of globules of mercury; and sometimes there is a chalk stone of the earthy carbonates. The nucleus is mostly composed of a little mucus from the gall-bladder. The physical characters are varied, some being white, simple, and homogeneous; but more commonly they are mixed, either in radiations from the nucleus or in concentric rings around it. The crust is nearly always of pure cholesterin, but sometimes it is formed of fatty acids, coloring matter, and cholesterin.

*Etiology.*—The oldest supposable cause for the formation of gall-stone is inspissation, but it is rare to find all the constituents of the bile, except water, in a single stone. Other theories are, lessened secretion of sodium, action of an acid; increased amount of lime, forming with the pigments a nucleus; secretion of calcium from the mucous membrane of the gall-bladder. All stones, however, do not contain all these substances. Formation is chiefly due to the precipitation of some one substance, and this does not occur until the glycocholate or taurocholate of sodium decomposes, when the reaction is changed to acid. In order that the concretions should be of any size, it is necessary for the bile to be retained in the gall-bladder or ducts for some time. Sometimes erosions are formed in the stones, caused occasionally by depositions; sometimes they divide, and these conditions make their disposal easier of accomplishment.

The influence of age on the formation of biliary calculi is difficult to understand, unless it be due to the habit and changes age brings. They are most common over twenty-five years. Females are more prone than males, three to two. Multiparity predisposes, as do diseased conditions anywhere; morbid changes in the liver and its passages, cancer, adhesions to various organs. Another cause is sedentary habits. Too long intervals between meals favors stagnation. It is doubtful if there exists a diathesis predisposing to stone.

